# Flecainide-induced pneumonitis: a case report

**DOI:** 10.1186/s13256-022-03619-w

**Published:** 2022-11-02

**Authors:** Gauthier Moureau, Elies Zarrouk, Delphine Hoton, Franck Saint-Marcoux, Lidvine Boland, Vincent Haufroid, Philippe Hantson

**Affiliations:** 1 Department of Intensive Care, Cliniques St-Luc, Université catholique de Louvain, Avenue Hippocrate, 10, 1200 Brussels, Belgium; 2 Service de Pharmacologie, Toxicologie et Pharmacovigilance, CHU de Limoges, 87042 Limoges, France; 3 Department of Pathology, Cliniques St-Luc, Université catholique de Louvain, 1200, Brussels, Belgium; 4 Department of Clinical Chemistry, Cliniques St-Luc, Université catholique de Louvain, 1200, Brussels, Belgium; 5 Louvain Centre for Toxicology and Applied Pharmacology, Université catholique de Louvain, 1200, Brussels, Belgium

**Keywords:** Flecainide, Pneumonitis, Acute fibrinous and organizing pneumonia, Corticosteroids

## Abstract

**Background:**

We report a case of acute respiratory distress associated with a histological pattern of acute fibrinous and organizing pneumonia, and discuss the possible responsibility of flecainide therapy.

**Case presentation:**

A 61-year-old African woman developed a rapidly progressive dyspnea and required admission in the intensive care unit for orotracheal intubation and mechanical ventilation. Chest X-ray examination revealed bilateral infiltrates predominating in the basal part of both lungs. Lung computed tomography disclosed bilateral ground-glass opacities and septal thickening. After exclusion of the most common causes of infectious or immune pneumonia, a toxic origin was investigated and flecainide toxicity was considered. Lung biopsy was consistent with the unusual pattern of acute fibrinous and organizing pneumonia. Clinical and radiological improvement was noted after corticosteroid therapy, but the patient died from septic complications.

**Conclusion:**

Flecainide-induced lung injury has rarely been reported in the literature and remains a diagnosis of exclusion. The histological pattern of acute fibrinous and organizing pneumonia has been previously observed with amiodarone. There are no firm guidelines for the treatment of acute fibrinous and organizing pneumonia, but some patients may positively respond to corticosteroids.

## Background

Drug-induced lung injury (DILI) remains a challenging diagnosis in critically ill patients, and usually requires a careful exclusion of more common causes such as infections, granulomatous diseases, idiopathic interstitial pneumonia, autoimmunity, and other miscellaneous disorders [1]. Pulmonary toxicity of flecainide is uncommon but often under-recognized. Clinical presentation may vary from acute pneumonitis to subacute or chronic manifestations such as interstitial lung disease or diffuse alveolar damage [2].

## Case presentation

**Fig. 1:**
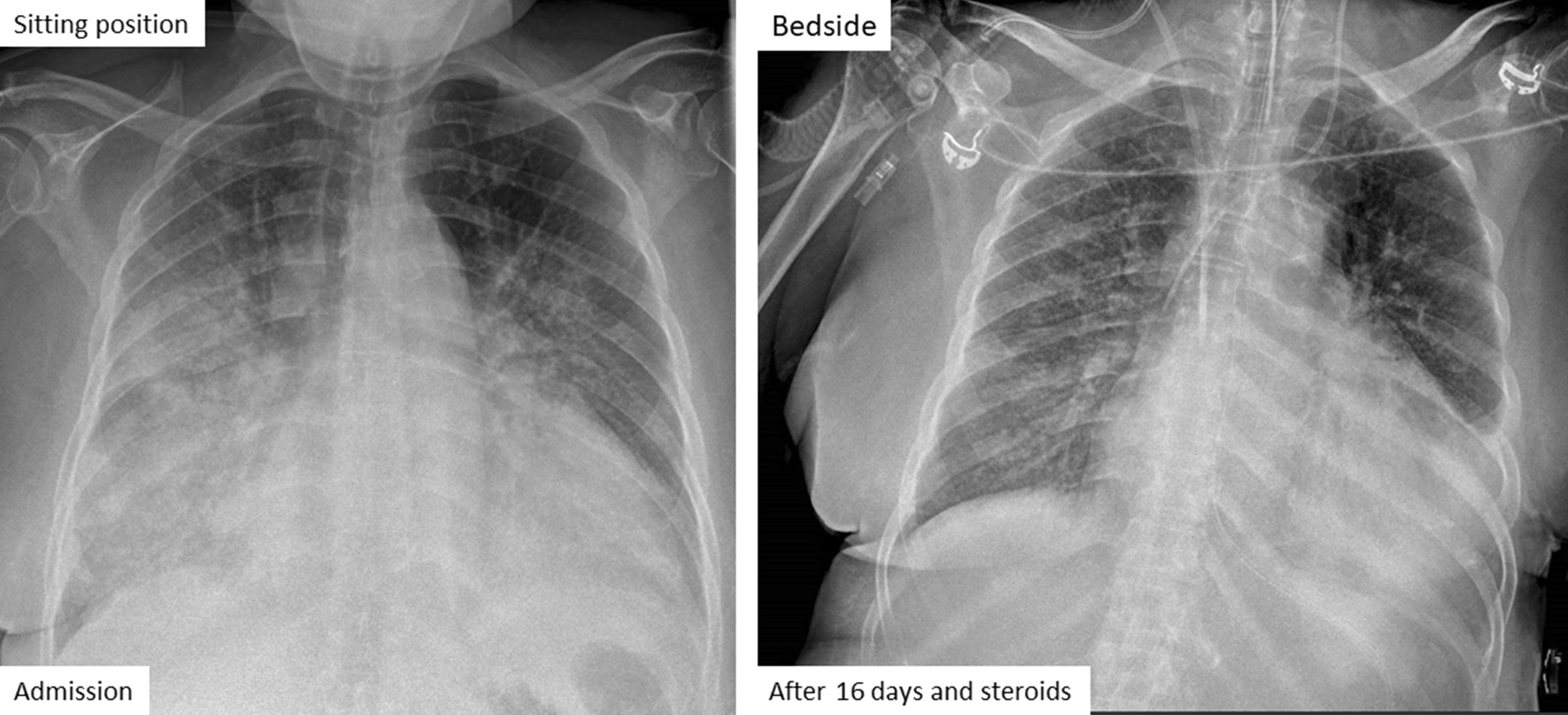
Chest X-ray examination on admission (left) and 16 days after the start of corticosteroids (right)

**Fig. 2:**
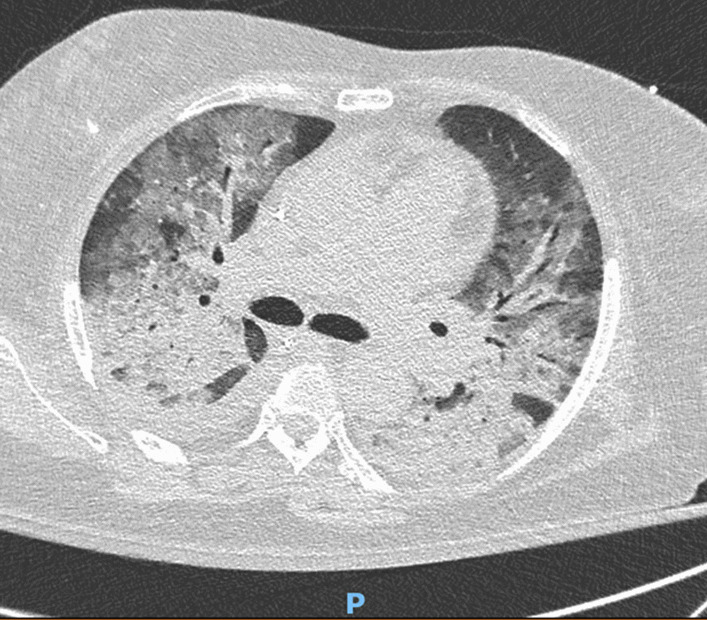
Lung computed tomography on ICU admission, with diffuse ground-glass opacities and septal thickening

**Fig. 3:**
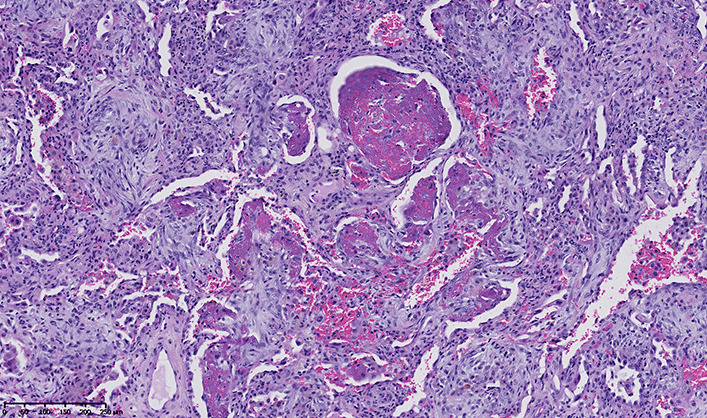
Microscopic examination of lung biopsy. Hemalun-eosine-safran coloration. ×10 magnification. Pattern consistent with acute fibrinous and organizing pneumonia: fibrinous exudate is mixed with the fibroblast plugs within air spaces.

A 61-year-old African woman (68 kg weight) was referred to the intensive care unit for a rapidly progressive dyspnea and dry cough. Her medical past history was marked by a kidney transplantation in 2001 for nephroangiosclerosis, along with malignant hypertension. Current medications included amlodipine, bisoprolol, lisinopril, tacrolimus, prednisolone (3 mg/day), linagliptine, and rosuvastatin. Since 28 months, she was also receiving flecainide (100 mg once daily) for recurrent supraventricular tachyarrhythmia. In the emergency department, her body temperature was 36.7°C, respiratory rate 34/min, heart rate 66/min, arterial pressure 131/48 mmHg, and pulsed oxygen saturation (SpO_2_) 67%. Lung auscultation revealed bilateral crackles in the lower lobes. Relevant laboratory investigations showed C-reactive protein (CRP) of 375.1 mg/L (< 5.0 mg/L), creatinine of 4.49 mg/dL (0.60–1.30 mg/dL), white blood cell count of 24,720.10^3^/µL, and lactate dehydrogenase (LDH) of 508 IU/L (< 250 IU/L ). Arterial blood gas analysis revealed a pH of 7.41, pO_2_ of 57 mmHg, pCO_2_ of 28 mmHg, total bicarbonate of 18 mmol/L, and lactate of 0.6 mmol/L. On chest X-ray examination, bilateral infiltrates predominated in the basal part of both lungs (Fig. 1). Lung computed tomography (CT) disclosed bilateral ground-glass opacities and septal thickening (Fig. 2). Empirical antimicrobial therapy was started with piperacillin/tazobactam and sulfamethoxazole/trimethoprim. Antihypertensive medications and flecainide were withdrawn. Because of the rapidly progressive hypoxemia, oral intubation was required for mechanical ventilation with inspired fraction of oxygen (FiO_2_) 0.80. Before antimicrobial therapy, a sputum analysis from the endotracheal aspirate failed to reveal any microorganism on direct examination, and culture remained sterile. Continuous renal replacement therapy (CRRT) was also temporarily needed. Examination and cultures of bronchoalveolar lavage (BAL; 73% neutrophils, 16% macrophages) were negative for mycobacteria (including atypical), bacteria, fungi, parasites and virus (multiplex PCR respiratory panel). In the serum, PCR was negative for cytomegalovirus (CMV), and the Aspergillus galactomannan test was negative. Immunofluorescent staining and PCR were negative on BAL for *Pneumocystis jirovecii*. Screening for myositis-specific antibodies (and also other autoimmune disorders) was negative. Echocardiography confirmed hypertrophic cardiomyopathy and aortic valve disease, with preserved left ventricle ejection fraction, but with a moderate to severe pulmonary hypertension. An open lung biopsy was performed on intensive care unit (ICU) day 4. Intravenous methylprednisolone was started (80 mg, corresponding to 1.25 mg/kg) for 11 days, and was then shifted to 64 mg prednisolone orally. Antimicrobial therapy was definitely stopped on ICU day 6. Microscopic examination revealed a diffuse filling of alveolar spaces by fibrin balls associated with organizing pneumonia (Fig. 3). Culture of lung tissue remained sterile. Genotyping for cytochrome P450 2D6 was also performed and the patient was *2/*4 with a duplication for *4. This implies that the patient may be considered as an intermediate metabolizer for flecainide. Extubation was possible on ICU day 15. There was also a significant radiological improvement (Fig. 1). Unfortunately, the recent episode of marked hypoxemia worsened the preexisting pulmonary hypertension and the patient died on day 21 from a mixed cardiogenic and septic shock following *Candida albicans* bacteremia.

### Determination of flecainide concentration in plasma and lung tissue

Plasma and lung tissue samples were obtained 4 days after flecainide withdrawal. The plasma flecainide concentration was 32.3 µg/L. This measurement was obtained from liquid chromatography coupled to high resolution mass spectrometry (LC-HRMS ) using an LCMS-9030 system (Shimadzu Corporation, Japan). The method presented a limit of detection of 1 µg/L. In the lung tissue, flecainide concentration was 1.6 µg/g of tissue. This measurement was performed using a liquid chromatography coupled to diode array detection (LC-DAD).

## Discussion and conclusions

Flecainide-induced lung injury has rarely been reported in the literature [3–8]. Clinical presentation varied largely in the published cases. In our case, in a 70-year-old woman who was previously treated with amiodarone, the recent introduction of flecainide (two doses of 100 mg) resulted in an acute hypoxemic respiratory failure with diffuse ground-glass opacities bilaterally on the chest computed tomography (CT). After exclusion of hospital-acquired pneumonia by a large infectious workup of common infectious causes, the patient received empiric intravenous methylprednisolone therapy (1 mg/kg/day) and her respiratory status and oxygen requirement improved over the next 4 days [3]. Subacute presentation has also been reported in patients who were treated with flecainide for several months or years. In an 80-year-old woman (50 mg flecainide twice daily for 4 years), a presumptive diagnosis of flecainide-induced organizing pneumonia led to the withdrawal of flecainide and to the prescription of prednisone, with a rapid improvement in the patient’s symptoms [7]. In two other cases, a 73-year-old man and a 75-year-old man (4 and 22 months treatment duration, respectively) had symptoms of low-grade fever, dry cough, and breathlessness, and bilateral ground-glass opacities were seen on chest CT [4]. When extensive examination and cultures of bronchoalveolar lavage (BAL) or lung tissue were negative, flecainide was stopped and after initiation of prednisone (1 mg/kg), pneumonitis rapidly improved clinically and radiologically.

Flecainide-associated lung injury remains a diagnosis of exclusion, and our case is no exception to this rule. Among the antihypertensive drugs taken by the patient, bisoprolol, amlodipine, and lisinopril were also withdrawn, but these drugs do not cause lung interstitial disorders. Particularly in immunocompromised patients, exclusion of infectious etiologies is mandatory, and especially opportunistic infections. There is no specific feature on chest computed tomography (CT), even if reversible ground-glass opacities were described with other cases of amiodarone- or flecainide-associated acute lung injury. The chemical structure of flecainide appears different from that of tocainide or amiodarone, but these drugs share the property of lung accumulation following chronic use [8, 9]. The mechanism of injury could be a cell-mediated immunologic reaction [6]. Lymphocytic and eosinophilic inflammation was prominent in BAL (and in the lung biopsy of one patient) in the two patients described by Pesenti *et al.* [4]. A positive leukocyte migration inhibition test in the presence of flecainide has also been reported [6].

In patients given therapeutic doses of flecainide, plasma concentration is usually within a range of 200–1000 µg/L for trough levels. The average elimination half life is about 12 hours, but can be significantly increased in patients with renal or hepatic function impairment. We were not able to calculate the terminal elimination half life in our patient, but she presented a combination of acute renal failure and a genetic status of intermediate metabolizer for flecainide.

On the other hand, flecainide has been shown to accumulate significantly in lung tissue [8, 9]. After a 4-month course of flecainide (150 mg twice daily), a 60 year-old man died after the acute onset of dyspnea. At autopsy, extremely high concentrations of flecainide were found in the lung (143 µg/g wet weight), contrasting with the content of other tissues [9]. This observation was confirmed by another postmortem investigation in a 49-year-old woman who was prescribed flecainide (150 mg twice daily) for 1 year. Microscopic examination was consistent with organizing interstitial pneumonia. A low but detectable concentration of flecainide was found in lung tissue 66 days after withdrawal of the drug [8]. Like amiodarone and tocainide, flecainide seems to have a high affinity for lung tissue and toxicity could also be related to drug accumulation [10, 11].

In our observation, the cellularity of the BAL was not predominantly lymphocytic. Microscopic examination was consistent with the histologic pattern of acute fibrinous and organizing pneumonia (AFOP) [12]. Among the drugs that were potentially associated with AFOP, amiodarone was mentioned in the first description of this entity [13, 14]. Classically, AFOP exhibits a poor prognosis with up to 50% mortality [13]. However, the clinical course is also likely influenced by the underlying conditions and triggering factors. Patients with the subacute form of AFOP could positively respond to corticosteroids, provided that the treatment is started at an early stage.

## Data Availability

All supporting data can be obtained by direct correspondence with the corresponding author (PH).

## Abbreviations

AFOP: Acute fibrinous and organizing pneumonia
BAL: Bronchoalveolar lavage
CRRT: Continuous renal replacement therapy
CT: Computed tomography
DILI: Drug-induced lung injury
LC-DAD: Liquid chromatography coupled to diode array detection
LC-HRMS: Liquid chromatography coupled to high resolution mass spectrometry
